# Usability and User Experience of Cognitive Intervention Technologies for Elderly People With MCI or Dementia: A Systematic Review

**DOI:** 10.3389/fpsyg.2021.636116

**Published:** 2021-04-22

**Authors:** Leslie María Contreras-Somoza, Eider Irazoki, José Miguel Toribio-Guzmán, Isabel de la Torre-Díez, Angie Alejandra Diaz-Baquero, Esther Parra-Vidales, María Victoria Perea-Bartolomé, Manuel Ángel Franco-Martín

**Affiliations:** ^1^Faculty of Psychology, University of Salamanca, Salamanca, Spain; ^2^Department of Research and Development, Iberian Institute of Research in Psycho-Sciences, INTRAS Foundation, Zamora, Spain; ^3^Department of Signal Theory and Communications, University of Valladolid, Valladolid, Spain; ^4^Biomedical Research Institute of Salamanca, University of Salamanca, Salamanca, Spain; ^5^Río Hortega Hospital’s Psychiatry and Mental Health Service, Valladolid, Spain; ^6^Zamora Public Welfare Complex, Zamora, Spain

**Keywords:** cognitive intervention, technology, usability, user experience, dementia, MCI

## Abstract

**Introduction:**

Incorporating technology in cognitive interventions represents an innovation, making them more accessible, flexible, and cost-effective. This will not be feasible without adequate user-technology fit. Bearing in mind the importance of developing cognitive interventions whose technology is appropriate for elderly people with cognitive impairment, the objective of this systematic review was to find evidence about usability and user experience (UX) measurements and features of stimulation, training, and cognitive rehabilitation technologies for older adults with mild cognitive impairment (MCI) or dementia.

**Method:**

The Medline, PubMed, Scopus, ScienceDirect, and PsycINFO databases were searched for literature published in the last 10 years (2009–2019), and three researchers independently reviewed potentially eligible studies, following specific inclusion criteria. A systematic review of the studies was conducted, presenting a qualitative synthesis of usability and UX measures with their outcomes, study characteristics and features of the cognitive intervention technologies.

**Results:**

Ten studies were selected: five were cognitive stimulation and five were cognitive training. Most of them (60%) were computer-based programs with a serious game format. Efficiency and effectiveness were the most frequent measurements used for collecting objective usability data, showing that elderly people with cognitive impairment require more time (45%) and help (40%) but can complete tasks (60%). Regarding UX or subjective usability data, questionnaires and scales were the most used methods, reporting positive experience despite certain difficulties with the interface in five studies.

**Conclusion:**

Measuring usability and UX in cognitive intervention technologies for older adults with MCI or dementia provides an integrated view that can contribute to their development according to the needs and characteristics of the target population. More research is required to include this population group in usability and UX studies, as well as standardized tools and consensus on the relationship of these terms to guarantee the future effectiveness of cognitive intervention technologies.

**Review registration:**

This review was registered in the PROSPERO (CRD42020158147) International Register of Systematic Review Protocols.

## Introduction

It is currently estimated that every 3 s someone develops dementia, and the annual cost of dementia care is estimated at US $ 1 billion, a quantity that will double by 2030 (Alzheimer’s Disease International, 2019). Mild cognitive impairment (MCI) is often a transitional stage from normal aging’s cognitive decline to dementia, in which the functional abilities of daily life are not preserved (Petersen et al., 2014). As expected, studies have shown that people who undergo normal aging processes display better cognitive performance compared to elderly people with MCI and those with dementia, the latter group having the greatest difficulties (Lavrencic et al., 2019).

Cognition-based interventions are increasingly considered as an important complement and even an alternative to pharmacological treatments for people with dementia (Bahar-Fuchs et al., 2013). In the MCI population, cognitive interventions have been effective in optimizing cognitive functioning, reducing cognitive impairment and delaying the onset of dementia (Faucounau et al., 2010). There are three main approaches to cognition-focused interventions (Bahar-Fuchs et al., 2013): cognitive stimulation (CS), cognitive training (CT), and cognitive rehabilitation (CR).

Cognitive stimulation is usually used in groups for older adults with cognitive impairment, including a variety of activities to keep cognitive functions active in a general and entertaining way (Woods et al., 2012). It can also be used with healthy elderly individuals (HE) to prevent cognitive decline (Rosell, 2018). CT consists of guided standardized exercises to improve performance in certain cognitive functions (Kallio et al., 2017). It can be used in elderly with or without cognitive impairment (Ledreux et al., 2019), wither individually or in groups (Oltra-Cucarella et al., 2018). CR is an individualized approach aimed at improving the functionality in daily living of older adults with cognitive impairment, thus helping to reduce caregiver burden (Oltra-Cucarella et al., 2018; Germain et al., 2019).

Computer-based cognitive interventions have the advantage of being more accessible to the public at large, and are also flexible, self-administered and cost effective (Faucounau et al., 2010; Toribio-Guzmán et al., 2018). In addition, technology allows cognitive exercises to be presented in new and engaging ways (Kueider et al., 2012). For example, video games have moving images, sounds, and feedback that make them more attractive and rewarding than printed materials (Toril et al., 2014). Some authors use the term serious games for tools aimed at specific purposes, such as cognitive games designed to improve cognitive functions rather than for entertainment alone (Robert et al., 2014).

However, good user-technology fit is essential to prevent technology from being ignored or misused (Meiland et al., 2017). Hence, it is important that technologies have a human-centered design, taking users and usability into account in their development (International Organization for Standardization [ISO], 2019a). Usability is the degree to which a product, service or system can be used with effectiveness, efficiency and satisfaction by certain users in a specific context to achieve an objective (International Organization for Standardization [ISO], 2019b).

Different usability testing methods are available and can be used during all phases of a product’s development to ensure that its design can meet high-quality standards, identifying problems and correcting them for easy, efficient and effective user-system interaction (Toribio-Guzmán et al., 2017). Furthermore, usability also assesses satisfaction, which, in turn, involves user experience (UX) (International Organization for Standardization [ISO], 2019a). UX consists of the perceptions, emotions, beliefs, preferences and behaviors of the users that happen before, during and after the utilization of a product, service or system (International Organization for Standardization [ISO], 2019b). UX focuses on subjective, temporal, situated and holistic attributes, and on design and user interaction (Bargas-Avila and Hornbæk, 2011; Roto et al., 2011).

Nevertheless, usability issues in cognitive intervention technologies for people with dementia are scarcely mentioned in research (Meiland et al., 2017). Moreover, there are studies that do not provide an integrated understanding of UX in association with technological devices (Megges et al., 2018), and one study reported that few technology systems are specifically designed to approach the cognitive limitations that affect older adults with cognitive impairment (Wargnier et al., 2018). In a population sector that is already subject to the frustration and lack of confidence that is associated with the limitations of their condition, the impact of unsuitable technological designs can add to such negative feelings (Smeenk et al., 2018).

Technologies aimed at people with cognitive impairment have to take into account their needs, preferences, abilities and limitations, since lack of awareness of their particularities not only affects them, but also their families and society at large, involving a costly burden for the community (Czaja et al., 2019). Given the importance of developing cognitive interventions whose technology is acceptable, usable and relevant to the elderly population with cognitive impairment, the objective of this systematic review was to obtain evidence about usability and UX measures and features of stimulation, training, and cognitive rehabilitation technologies for older adults with MCI or dementia.

## Materials and Methods

### Materials

This systematic review focused on usability and UX studies that address stimulation, training, and cognitive rehabilitation technologies for older adults with MCI or dementia, seeking evidence regarding such cognitive intervention technologies’ usability and UX measures and characteristics.

The PRISMA (Preferred Reporting Items for Systematic reviews and Meta-Analyses) guidelines were followed to ensure the review’s transparency and clarity (Liberati et al., 2009). Accordingly, the analysis and presentation of quality evidence-based information allows it to be adequately conveyed to those interested in mental health support technological programs, whose fast and growing development means that not all of them include the characteristics that would be desirable or achieve suitable goals (Baumel, 2016; Baumel et al., 2017).

This review was registered in the PROSPERO (CRD42020158147)^1^ International Register of Systematic Review Protocols, whose purpose is also to increase transparency in systematic reviews, avoiding duplication and minimizing bias (Schiavo, 2019).

### Procedure

The PROSPERO website was searched for previous systematic reviews on the topic and none were found, which validated the purpose of this review. The Medline, PubMed, Scopus, ScienceDirect, and PsycINFO databases were searched in August and September 2019 using certain combinations of keywords to delimit the search (Table 1).

**TABLE 1:** Keywords combination.

Usability AND (tech* OR software OR computer) AND “cognitive stimulation” AND (dementia OR “mild cognitive impairment” OR MCI)
Usability AND (tech* OR software OR computer) AND “cognitive training” AND (dementia OR “mild cognitive impairment” OR MCI)
Usability AND (tech* OR software OR computer) AND “cognitive rehabilitation” AND (dementia OR “mild cognitive impairment” OR MCI)
(“user experience” OR UX) AND (tech* OR software OR computer) AND “cognitive stimulation” AND (dementia OR “mild cognitive impairment” OR MCI)
(“user experience” OR UX) AND (tech* OR software OR computer) AND “cognitive training” AND (dementia OR “mild cognitive impairment” OR MCI)
(“user experience” OR UX) AND (tech* OR software OR computer) AND “cognitive rehabilitation” AND (dementia OR “mild cognitive impairment” OR MCI)

ScienceDirect did not support the truncation symbol, so the term technology was used instead of tech^∗^. In all the databases, results were limited to the last 10 years (2009 – 2019) and to being written in English or Spanish. The search results were exported to the EndNote citation manager. A total of 552 studies were obtained, of which 305 remained after the removal of duplicates. The titles and/or abstracts of these studies were read, and the following criteria were used to find potentially eligible articles:

Inclusion criteria:

• People aged 60 and over with MCI (all subtypes) or with one of the following types of dementia: Alzheimer, frontotemporal dementia, vascular dementia
• Any type of technology mainly or partly aimed at stimulation, training or cognitive rehabilitation
• Stimulation, training, or cognitive rehabilitation technologies where measurements or characteristics of usability and/or user experience are provided
• Journal articles with descriptive, explanatory, experimental or analytical studies as well as clinical trials and pilot studies

Exclusion criteria:

• Older adults with other types of dementia or clinical conditions (Lewy Body, Pick’s disease, Creutzfeldt-Jakob disease, alcohol-related dementia, AIDS dementia complex, Huntington’s disease, Parkinson’s disease, Down syndrome, brain injury) or healthy older adults
• Programs where the use of technology was not intended for therapeutic purposes of stimulation, training or cognitive rehabilitation
• Stimulation, training or cognitive rehabilitation technologies with no description regarding usability or user experience
• Systematic/literature reviews, meta-analyses, editorials, newspapers, magazines, book chapters, and conference papers

**FIGURE 1:**
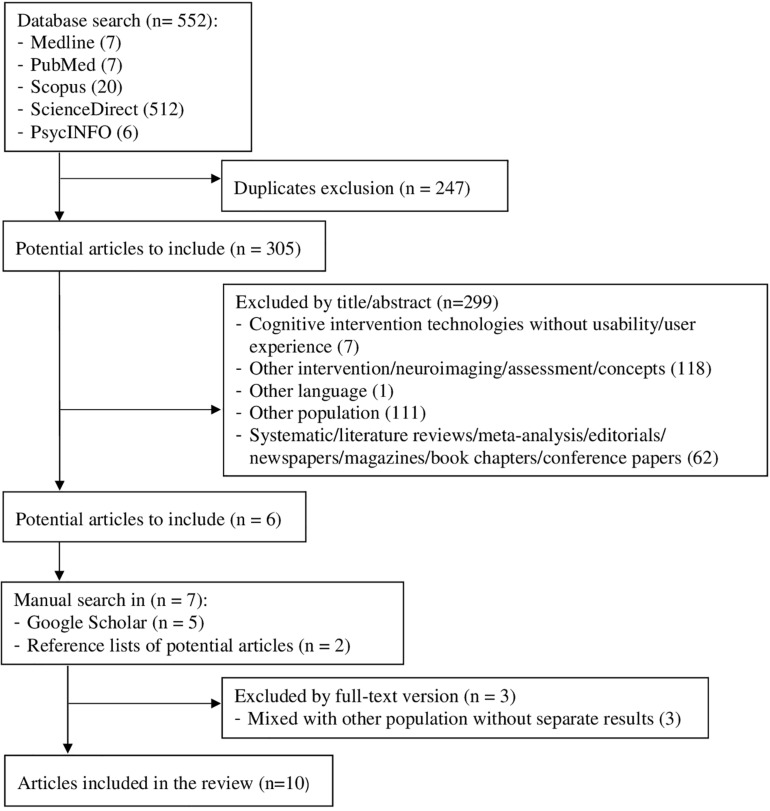
Search procedure and studies selection.

This narrowed the selection down to six articles. In order to find more potentially eligible studies, a manual Google Scholar search was conducted based on the terms ‘usability technology cognitive stimulation training rehabilitation dementia MCI.’ In addition, the reference lists of all the selected studies were screened to ensure that no possible articles were left out. These two steps broadened the sample to 13 articles, whose full-text versions were examined to verify whether they were appropriate for inclusion. Three researchers conducted this process independently, subsequently comparing their results to achieve a consensus on which studies to include or exclude. Finally, a total of 10 articles were included. This search and selection process is summarized in Figure 1.

Of the 10 included studies, that by Kyriazakos et al. (2017) addressed the target population and another sample that did not meet the inclusion criteria, but it was not excluded because the results obtained for the target population were presented separately.

### Data Extraction

The measures and features to be extracted and analyzed were chosen according to the main characteristics of usability; namely, effectiveness, efficiency and satisfaction (considering the latter as part of UX), as defined in International Organization for Standardization [ISO] (2019b):

• Effectiveness: accuracy and completeness with which users achieve specific goals
• Efficiency: resources used in relation to the results achieved, such as time, effort, materials and costs
• User experience: perceptions, emotions, beliefs, preferences, comfort, accomplishments, responses and behaviors that happen before, during and after use, as well as the extent to which the user’s needs and expectations are met (satisfaction)

Considering the ongoing scientific debate about the overlap between usability and UX, this study approaches UX as an extension of usability, since UX focuses on assessing subjective aspects such as satisfaction (Sauer et al., 2020).

It should be noted that no articles were excluded from this data collection. The analysis was conducted based on the number and type of studies, participants’ socio-demographic characteristics, types of cognitive interventions and technology used, measures and features of usability and UX and their main results.

### Data Analysis

Due to the different usability and UX measures, as well as the variety of methodologies used in the studies, a qualitative synthesis of the results was performed following the Cochrane guidelines for data synthesis and analysis (Ryan, 2013). Firstly, study characteristics such as design and participants are presented. Then, features such as type, format, software, and hardware of cognitive intervention technologies are reported. Finally, a description is given about the usability and UX measures found in the studies and their outcomes.

## Results

### Characteristics of the Studies

The search yielded 552 studies, narrowed down to six potential articles after the removal of duplicates and the screening of titles and abstracts according to the inclusion and exclusion criteria. Also based on these criteria, a manual search led to the addition of seven more potential studies. The full-text version of these 13 articles was read, and, finally, 10 articles were included for analysis (Figure 1).

The selected papers were eight pilot studies (González-Abraldes et al., 2010; Boulay et al., 2011; González-Palau et al., 2013; Manera et al., 2015; Djabelkhir et al., 2017; Garcia-Sanjuan et al., 2017; Kyriazakos et al., 2017; Tziraki et al., 2017) and two clinical trials (Haesner et al., 2015; Ben-Sadoun et al., 2016). Most of them had been conducted in Europe and the number of subjects that made up their samples ranged from 7 to 180, most of them women aged 60–90 with MCI or dementia (some with mild dementia and others with Alzheimer’s disease), as well as HE (Tables 2, 3).

**TABLE 2:** Participant’s sociodemographic characteristics.

Technology	Experimental group			Control group			Country
		
	Participants	Age (S.D.)	Sex	Participants	Age (S.D)	Sex	
**X-Torp** (Ben-Sadoun et al., 2016)	4 AD, 6 MCI	82.3 (±6.4)	4 W, 6 M	8 HE	71.4 (±10.1)	5 W, 3 M	France
**MINWii** (Boulay et al., 2011)	7 AD	88.5 (N/A)	4 W, 3 M	N/A	N/A	N/A	France
**CCS and CCE** (Djabelkhir et al., 2017)	10 MCI (CCS)	75.2 (±6.4)	7 W, 3 M	10 MCI (CCE)	78.2 (±7.0)	6 W, 4 M	France
**Tangibot** (Garcia-Sanjuan et al., 2017)	12 MCI, 12 SCI	81.33 (±8.48)	*32 W, 8 M	16 HE	81.33 (±8.48)	*32 W,8 M	Spain
**LLM** (González-Palau et al., 2013)	52 MCI, 33 MD	81.97 (±9.16) 83.44 (± 5.67)	64 W, 21 M	95 HE	81.87 (±6.84)	70 W, 25 M	Spain
**Web-based CT platform** (Haesner et al., 2015)	6 MCI	60–70 years (N/A)	3 W, 3 M	6 HE	>80 years	3 W, 3 M	Germany
**Telecognitio: two apps** (González-Abraldes et al., 2010)	8 MCI	75 (±6.7)	N/A	N/A	N/A	N/A	Spain
**eWALL** (Kyriazakos et al., 2017)	48: MCI ARI, COPD	Older adults (N/A)	N/A	N/A	N/A	N/A	Austria, Italy, Denmark, Netherlands
**Kitchen and Cooking** (Manera et al., 2015)	9 MCI, 12 AD	75.8 (±9.1) 80.3 (±6.3)	7 W, 2 M 8 W, 4 M	N/A	N/A	N/A	France
**Serious Game** (Tziraki et al., 2017)	24 Dementia	65 – 90 years (N/A)	15 W, 9 M	14 HE	65 – 90 years	11 W, 3 M	Israel

AD, Alzheimer disease; **apps**, applications; **ARI**, Age-Related Functional Impairments; **CCE**, computerized cognitive engagement; **CCS**, computer cognitive stimulation; **COPD**, chronic obstructive pulmonary disease; **CT**, cognitive training; **HE**, healthy elderly; **LLM**, long lasting memories; **M**, man; **MCI**, mild cognitive impairment; **MD**, mild dementia; **N/A**, not apply; **S.D.**, standard deviation; **SCI**, severe cognitive impairment; **W**, woman.

*, sex distribution on the entire sample.

**TABLE 3:** Methodological and descriptive characteristics of the studies.

		Intervention					

Technology	Design	Type	Format	Familiarization	Total duration	Dropouts	Other features
**X-Torp** (Ben-Sadoun et al., 2016)	Clinical trial	CS	Serious game	2 wks	1 mo (20-80 min/sess, 3 sess/wk)	N/A	Physical training
**MINWii** (Boulay et al., 2011)	Pilot study	CS	Serious game	1 sess	3 mos (10-20 min/sess, 1 sess/wk)	MP (n = 1); RTC after 2nd sess (*n* = 1)	Music therapy
**CCS and CCE** (Djabelkhir et al., 2017)	Pilot study	CCS	Exercises	1 sess	3 mos (90 min/sess, 1 sess/wk)	MP (*n* = 1, CCS)	CCS: social interaction CCE: training to use a tablet-PC, social interaction
**Tangibot** (Garcia-Sanjuan et al., 2017)	Pilot study	CT	Serious game	Before each task	1 sess (3 tasks/4 times each one)	RTT (n = 4); RTC after 1st contact (n = 2)	N/A
**LLM** (González-Palau et al., 2013)	Pilot study	CT	Exercises	N/A	3 mos (40 min/sess, 3 sess/wk)	MP (*n* = 7); FP (*n* = 4); RTC (*n* = 3)	Physical training
**Web-based CT platform** (Haesner et al., 2015)	Clinical trial	CT	Exercises	1 sess	1 sess	N/A	Social media
**Telecognitio: two apps** (González-Abraldes et al., 2010)	Pilot study	CS	Exercises	Before the intervention	4 sess (15 min/each app)	N/A	N/A
**eWALL** (Kyriazakos et al., 2017)	Pilot study	CT	Serious games	1 sess	6 wks	N/A	AAL; ELEs; AmI
**Kitchen and Cooking** (Manera et al., 2015)	Pilot study	CT	Serious games	1 sess	1 mo (subjects played it as much as they wanted)	RTC after first wk (*n* = 1)	N/A
**Serious Game** (Tziraki et al., 2017)	Pilot study	CS	Serious games	N/A	10 wks (20–30 min/sess, 1–2 sess/wk)	MP (*n* = 9); GEU (*n* = 3)	N/A

*AAL, active assisted living; **AmI**, ambient intelligence; **apps**, applications; **CCE**, computerized cognitive engagement; **CCS**, computerized cognitive stimulation; **CS**, cognitive stimulation; **CT**, cognitive training; **CT**, cognitive training; **ELEs**, enhanced living environments; **FP**, family problems; **GEU**, game easy and uninteresting; **LLM**, long lasting memories; **min**, minutes; **mo**, month; **mos**, months; **MP**, medical problems; **N/A**, not apply; **RTC**, refused to continue; **RTT**, refused to try; **sess**, session; **wk**, week; **wks**, weeks.*

The length of the interventions varied between a single session and 3 months. Sessions were given 1–3 times per week, and each of them lasted between 10 and 90 min. At the beginning, eight of the studies included a trial period for older adults to adapt to the tool (González-Abraldes et al., 2010; Boulay et al., 2011; Haesner et al., 2015; Manera et al., 2015; Ben-Sadoun et al., 2016; Djabelkhir et al., 2017; Garcia-Sanjuan et al., 2017; Kyriazakos et al., 2017) that ranged between 1 session and 2 weeks (Table 3).

There were dropouts reported in six of the studies (Boulay et al., 2011; González-Palau et al., 2013; Manera et al., 2015; Djabelkhir et al., 2017; Garcia-Sanjuan et al., 2017; Tziraki et al., 2017), with rates ranging from 4.8 to 31.6%. The most frequent reasons for withdrawal were medical problems (50%), followed by reluctance to continue after the first contact with the technological tool (19.5%), family problems as well as refused to try (11.1% respectively) and, lastly, because they considered the exercise easy or uninteresting (8.3%) (Table 3).

### Features of Cognitive Intervention Technologies

Regarding the type of cognitive intervention, five were CS technologies: X-Torp (Ben-Sadoun et al., 2016), MINWii (Boulay et al., 2011), Computer Cognitive Stimulation (CCS) (Djabelkhir et al., 2017), Telecognitio (González-Abraldes et al., 2010) and Serious Game (Tziraki et al., 2017); and another five were aimed at CT: Tangibot (Garcia-Sanjuan et al., 2017), Long Lasting Memories (LLM) (González-Palau et al., 2013), eWALL (Kyriazakos et al., 2017), Kitchen and Cooking (Manera et al., 2015), and Web-based CT platform (Haesner et al., 2015). This last study and two others (González-Abraldes et al., 2010; Djabelkhir et al., 2017) were different from the rest because they used technology that already existed to gather information or develop their own. On the other hand, none of the studies included technology aimed at CR (Table 3).

In four of the studies the cognitive intervention technologies found consisted of cognitive exercises (González-Abraldes et al., 2010; González-Palau et al., 2013; Haesner et al., 2015; Djabelkhir et al., 2017) and six studies were based on serious games (Boulay et al., 2011; Manera et al., 2015; Ben-Sadoun et al., 2016; Garcia-Sanjuan et al., 2017; Kyriazakos et al., 2017; Tziraki et al., 2017). It should be noted that the games or video games whose purpose was linked to cognitive intervention rather than entertainment were serious games (Robert et al., 2014). On the other hand, several were programs with other functions such as physical training, social interaction, music therapy and assisted environments (Table 3).

The most commonly used hardware for cognitive interventions was the personal computer (60%), followed by the Tablet (40%), touch screens or screens (30%, respectively), gamepad/joystick, sensor or smartphone (20%, respectively). The least used were robots, mouse and headphones (10%, respectively). The described characteristics of the software and hardware were those strictly used and mentioned in the cognitive area of the studies found (Table 4).

**TABLE 4:** Hardware and software of the studies’ cognitive interventions.

	Hardware										Software		
		
Technology	PC	Tablet	Smart	Robot	Mouse	Screen	Touch	Sensor	Gamepad, Joystick, other	Head	Program	App	Web
			phone				screen			phones			platform
**X-Torp** (Ben-Sadoun et al., 2016)	**Yes**	No	No	No	No	**Yes**	No	**Yes**	No	No	**Yes**	No	No
**MINWii** (Boulay et al., 2011)	**Yes**	No	No	No	No	**Yes**	No	No	**Yes**	No	**Yes**	No	No
**CCS** (Djabelkhir et al., 2017)	No	**Yes**	No	No	No	**Yes**	No	No	No	No	No	No	**Yes**
**Tangibot** (Garcia-Sanjuan et al., 2017)	No	No	**Yes**	**Yes**	No	No	No	**Yes**	**Yes**	No	**Yes**	No	No
**LLM** (González-Palau et al., 2013)	**Yes**	No	No	No	No	No	**Yes**	No	No	**Yes**	**Yes**	No	No
**Web-based CT platform** (Haesner et al., 2015)	**Yes**	**Yes**	**Yes**	No	No	No	No	No	No	No	No	No	**Yes**
**Telecognitio: app A and B** (González-Abraldes et al., 2010)	Yes ***	No	No	No	Yes **	No	Yes *	No	No	No	No	Yes ***	No
**eWALL** (Kyriazakos et al., 2017)	**Yes**	No	No	No	No	No	**Yes**	No	No	No	No	**Yes**	No
**Kitchen and Cooking** (Manera et al., 2015)	No	**Yes**	No	No	No	No	No	No	No	No	**Yes**	No	No
**Serious Game** (Tziraki et al., 2017)	No	**Yes**	No	No	No	No	No	No	No	No	**Yes**	No	No

CCS, computerized cognitive stimulation; CT, cognitive training; LLM, long lasting memories; PC, personal computer.

*, App A; **, App B; ***, Both apps.

### Measures of Usability and UX in Cognitive Intervention Technologies

To facilitate understanding and comparison of the measures found, they were divided into usability and UX, considering UX as an extension of usability that focuses on subjective data. The measures are shown in Table 5.

**TABLE 5:** Usability and UX measures and results of studies in cognitive intervention.

Technology	Usability		UX	
		
	Measures	Results	Measures	Results
**X-Torp** (Ben-Sadoun et al., 2016)	Completed tasks	HE > AD, MCI	TAM: Ease of use	-Difficulty: AD, MCI > HE (nss) -Competence: HE > AD, MCI (it increased for all)
	Time to complete tasks	HE < AD, MCI	TAM: Usefulness	Interest: AD, MCI > HE
			PANAS	Positive emotions: HE > AD and MCI
			Time spent doing it*	No differences between groups
**MINWii** (Boulay et al., 2011)	Errors	4 participants decreased tasks errors	Satisfaction Questionnaire	High satisfaction of all participants
	Time to complete tasks	6 participants decreased the time Everyone held the joystick correctly	Verbalizations and behaviors	Participants expressed a desire to continue
	Interventions by moderator	Verbal interventions decreased Few physical interventions were made		
**CCS and CCE** (Djabelkhir et al., 2017)	N/A	N/A	Attendance rates	Everyone attended all sessions
			Motivation scale	High levels before and after the intervention by all
			Interviews	Both groups: -Main motivations: resist AD onset and loneliness -Group sessions considered engaging and stimulating -Expressed a desired to continue on a regular basis
**Tangibot** (Garcia-Sanjuan et al., 2017)	Completed tasks	HE > MCI (nss) MCI > SCI	Verbalizations and behaviors	62.5% of subjects showed enjoyment: HE > MCI, SCI
	Time to complete tasks	HE < MCI SCI ≈ MCI	Questions about whether they liked it - interview	80% of participants liked it: HE > MCI, SCI
	Errors	SCI > MCI, HE HE > MCI (nss)		
	Unnecessary actions	HE < MCI		
	Interventions by moderator	57.5% of participants needed help: SCI > MCI, HE		
**LLM** (González-Palau et al., 2013)	Interventions by moderator	Needed explanations: PWD, MCI > HE	Satisfaction scale	No differences between groups: -73.0% it met their expectations -66.9% felt confident using technologies -83.7% found it beneficial to health
			Affective scale	No differences between groups: 79.0% had fun
			Sustainability scale	No differences between groups: -78.1% thought it would be worth paying -84% expressed a desire to continue -96.1% would recommend it
			Ease of use and learn scale	-60.1% of participants found it easy to learn -40% of PWD found it harder to use without help
**Web-based CT platform** (Haesner et al., 2015)	N/A	N/A	Interviews	MCI subjects would prefer audio-video communication, but not with strangers HE would prefer messages or emails MCI > HE: preference to sessions outside of a classroom environment, have adapted levels of difficulty, receive personal feedback Both groups would like to: Use it on a regular basis for cognitive health Repeat exercises as often as they wished Have a variety of playful exercises Have a preliminary and subsequent progress tests Have not potential distractions: loud noises, bright colors, lots of animations Have cognitive self-educational supplement Have background information about other users Personal data to be handled confidentially Be run by a trusty church, government, medical services
			Technology Commitment Questionnaire	HE > MCI: 44.8/60 and 35.5/60 respectively
**Telecognitio: app A and B** (González-Abraldes et al., 2010)	N/A	N/A	*Ad hoc* questionnaire	Exit icon difficulty: A > B, 50% and 37.5% respectively Difficulty retaining questions: A > B, 100% and 37% respectively Hardware difficulty: A ≈ B, 62.5% accuracy and pulse time errors on touch screen and 62.5% handling the mouse, respectively App A: 62.5% pause icon difficulty 62.5% considered “very little” the time to respond
**eWALL** (Kyriazakos et al., 2017)	N/A	N/A	UEQ	MCI participants preferred cognitive exercise, activity and sleep apps
			TAM	
**Kitchen and Cooking** (Manera et al., 2015)	Completed tasks	70% successfully completed	Satisfaction scale	High satisfaction: AD > MCI
			Interest scale**	It interested them
			Motivation scale**	Intrinsic motivation > external motivation
			PANAS	Positive emotions > negative emotions
			PFS	Not very fatigued
			Time spent doing it*	3h 48 min at home
			Tasks done*	85% done at home
**Serious Game** (Tziraki et al., 2017)	Completed tasks	61% completed correctly by PWD. Auditory cueing improved their performance	Verbalizations and behaviors	HE: Found it too easy and not highly engaging PWD: Expressed fun and engaging. Auditory cueing improved their engagement Increased their self-efficacy Interacted and spoke to the tablet Remembered certain easy and difficult components Developed learning techniques
	Time to completed tasks	HE < PWD		

**AD**, Alzheimer disease; **app**, application; **CCE**, computerized cognitive engagement; **CCS**, computerized cognitive stimulation; **CT**, cognitive training; **h**, hour; **HE**, healthy elderly; **LLM**, long lasting memories; **MCI**, mild cognitive impairment; **min**, minutes; **N/A**, not apply; **nss**, not statistically significant; **PANAS**, positive affect negative affect scale; **PFS**, piper fatigue scale; **PWD**, people with dementia; **SCI**, severe cognitive impairment; **TAM**, technology acceptance model; **UEQ**, user experience questionnaire; **UX**, user experience.

*, participants were free to do the tasks as much as they wanted.

**, adapted from Gourlan et al. (2013).

Usability was measured using five main tools: number of completed tasks, number of errors or failed actions, time to complete tasks, number of unnecessary actions, and number of interventions made by the moderator. The first two corresponded to effectiveness, while the last three belonged to efficiency. On the other hand, six tools for UX measurement were found: questionnaires and scales, verbalizations and behaviors, attendance rates, interviews, time spent doing the activity and number of tasks completed. Only one study (González-Abraldes et al., 2010) used a single tool, while the other nine studies (Boulay et al., 2011; González-Palau et al., 2013; Haesner et al., 2015; Manera et al., 2015; Ben-Sadoun et al., 2016; Djabelkhir et al., 2017; Garcia-Sanjuan et al., 2017; Kyriazakos et al., 2017; Tziraki et al., 2017) used a combination of them.

Specifically, in terms of effectiveness, the most commonly used measure was the number of tasks completed by participants, found in four studies (Manera et al., 2015; Ben-Sadoun et al., 2016; Garcia-Sanjuan et al., 2017; Tziraki et al., 2017), followed by the number of errors, in two studies (Boulay et al., 2011; Garcia-Sanjuan et al., 2017). Regarding efficiency, the most frequent measure was the time it took to complete tasks, which appeared in four studies (Boulay et al., 2011; Ben-Sadoun et al., 2016; Garcia-Sanjuan et al., 2017; Tziraki et al., 2017), followed by moderator interventions, in 3 studies (Boulay et al., 2011; González-Palau et al., 2013; Garcia-Sanjuan et al., 2017) and the number of unnecessary actions in only 1 (Garcia-Sanjuan et al., 2017).

The measures mentioned above correspond to objective usability data collection. However, because usability also involves measuring the UX parameter of satisfaction, which involves collecting subjective data (Sauer et al., 2020), the following UX measures were considered in this review: participants’ verbalizations and behaviors, which appeared in three studies (Boulay et al., 2011; Garcia-Sanjuan et al., 2017; Tziraki et al., 2017), and interviews, also found in three studies (Haesner et al., 2015; Djabelkhir et al., 2017; Garcia-Sanjuan et al., 2017). These were followed by time participants spent on doing the tasks when there was no time limit, which was addressed in two studies (Manera et al., 2015; Ben-Sadoun et al., 2016); attendance rates, considered in one (Djabelkhir et al., 2017); and number of tasks that participants performed when free do as many as they wanted (regardless of whether they were poorly or well executed), also found in only one (Manera et al., 2015).

Questionnaires and scales were the most widely used tools for UX, appearing in eight studies (González-Abraldes et al., 2010; Boulay et al., 2011; González-Palau et al., 2013; Haesner et al., 2015; Manera et al., 2015; Ben-Sadoun et al., 2016; Djabelkhir et al., 2017; Kyriazakos et al., 2017). The standardized tools found were: Technology Acceptance Model (TAM), Positive Affect Negative Affect Scale (PANAS), User Experience Questionnaire (UEQ), Piper Fatigue Scale (PFS) and Technology Commitment Questionnaire. The non-standardized tools that were used tended to be Likert-type scales of satisfaction, motivation, affectivity, sustainability, interest, ease of use and learning.

The most used in UX were satisfaction scales, found in three studies (Boulay et al., 2011; González-Palau et al., 2013; Manera et al., 2015), followed by TAM (Ben-Sadoun et al., 2016; Kyriazakos et al., 2017), PANAS (Manera et al., 2015; Ben-Sadoun et al., 2016), and Motivation Scale (Manera et al., 2015; Djabelkhir et al., 2017) in two studies, respectively. Finally, UEQ (Kyriazakos et al., 2017), PFS (Manera et al., 2015), Technology Commitment Questionnaire (Haesner et al., 2015), Affective Scale (González-Palau et al., 2013), Sustainability Scale (González-Palau et al., 2013), Ease of Use and Learn Scale (González-Palau et al., 2013), Interest Scale (Manera et al., 2015), and an *ad hoc* questionnaire (González-Abraldes et al., 2010) were registered in one study, respectively.

**FIGURE 2:**
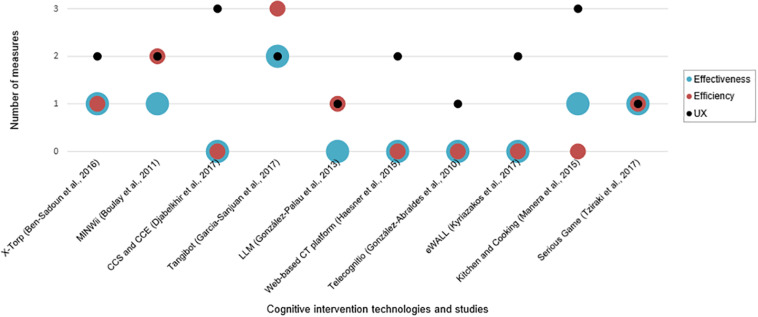
Number of effectiveness, efficiency, and UX measures used in the studies.

Of the 10 studies, 6 measured both usability (effectiveness and efficiency) and UX (Boulay et al., 2011; González-Palau et al., 2013; Manera et al., 2015; Ben-Sadoun et al., 2016; Garcia-Sanjuan et al., 2017; Tziraki et al., 2017), while four focused on UX (González-Abraldes et al., 2010; Haesner et al., 2015; Djabelkhir et al., 2017; Kyriazakos et al., 2017), as can be seen in Figure 2.

### Outcomes of Usability and UX Measures in Cognitive Intervention Technologies

The measurements found in the studies collected both objective and subjective data, so it is important and interesting to observe the differences or similarities in the results obtained from usability and UX measures. These results are summarized in Table 5.

#### Cognitive Stimulation Technologies

Djabelkhir et al. (2017) presented a CCS program that consisted of cognitive exercises and social interaction, as well as a Computerized Cognitive Engagement (CCE) system that involved training to use a tablet-PC and social interaction. Only UX measures were taken, obtaining similar positive results in both groups of older adults with MCI: everyone attended every session and high levels of motivation were reported before and after the interventions. The main motivations were to resist the onset of Alzheimer’s disease and to cope with loneliness. In addition, participants generally found the group sessions engaging and stimulating and expressed a desire to continue on a regular basis.

In the study by González-Abraldes et al. (2010), CS was also performed through cognitive exercises. They introduced two computerized apps for older adults with MCI. The differences were that app A had visual and audio statements, pictures, limited response time and a touch screen; while app B had audio statements, real pictures, no fixed response time, different levels of difficulty and a mouse. Only UX was measured. Both groups reported difficulties in using the exit icon and in remembering the questions to answer them, although those who used app A found more difficulty in both aspects (50 and 100%, respectively). In addition, the app A group reported difficulties associated with the pause icon and stated that there was “very little” time to answer (62.5% respectively). Finally, participants defined the use of the devices as complex: 62.5% of the participants in app A considered that they had to press the touch screen for too long or that it was inaccurate, while 62.5% of the subjects in app B found it difficult to use the mouse.

Tziraki et al. (2017) engaged in CS via serious games for older adults with dementia and HE. Usability results were positive and, although it took people with dementia longer to complete the tasks, they were able to complete 61% of them and their performance improved with listening cues. Positive results were also obtained in UX for people with dementia, who reported that the games were fun and engaging, listening cues contributing to the latter. In addition, they interacted and spoke to the tablet showing an increase in their self-efficacy, remembering easy and difficult components and developing learning techniques. However, HE found the tool too easy and hardly engaging.

According to the results of X-Torp (Ben-Sadoun et al., 2016), a serious game format with cognitive and physical training, people with Alzheimer’s disease and MCI completed fewer tasks and spent more time on them as compared to HE. UX results were consistent with those of usability, as adults with cognitive impairment reported more difficulties (although the results were not significant) and feelings of lack of competence (which increased for everyone). In general, the groups accepted the tool in terms of interest and positive emotions with the difference that people with Alzheimer’s disease showed more interest, while HE presented more positive emotions. Furthermore, there was no difference in the time spent by these groups on X-Torp.

As for MINWii (Boulay et al., 2011), which provided CS through a video game and music therapy, all the subjects with Alzheimer’s disease held the joystick correctly, 57.1% of them made fewer mistakes and 85.7% took less time to complete the tasks. Few physical and verbal moderator interventions were required. This positive degree of usability was consistent with UX, because participants were generally very satisfied and expressed their desire to continue using it.

#### Cognitive Training Technologies

In LLM (González-Palau et al., 2013), CT was performed via cognitive exercises to be completed by older adults with cognitive impairment (MCI and dementia) and HE. It also included a physical training function. In terms of usability, subjects with cognitive impairment were found to need more moderator explanations than HE. The perception of some people with dementia coincided with this fact in their UX, as 40% of them found it harder to use without help. However, 60.1% of participants found it easy to learn in general. In addition, the fact that 79.0% of the participants had fun and felt satisfied proved high acceptability of this tool: 73.0% reported that it met their expectations, 66.9% felt confident using technologies, and 83.7% found it beneficial for their health. There were also positive results in sustainability, as 78.1% thought it would be worth paying for it, 84% expressed a desire to continue using it and 96.1% would recommend LLM.

Haesner et al. (2015) used existing CT platforms and social media with older adults with MCI and HE to gather information about their preferences to develop their own web-based CT platform with cognitive exercises. Only UX measurements were taken. Both groups agreed that they would like to use it regularly for cognitive health, repeat the exercises as often as they wished, have a variety of playful exercises, have a preliminary and subsequent progress tests, have no potential distractions (such as loud noises, bright colors or too many animations), have a cognitive self-educational supplement, have background information about other users, have personal data handled confidentially and have the platform run by a trusted institution (church, government, or medical services). On the other hand, the differences between the groups were that HE preferred messages or emails, while MCI subjects preferred audio-video communication, although not with strangers. Likewise, the MCI group expressed a preference for sessions outside a classroom environment and adapted levels of difficulty and would like to receive personal feedback. However, HE showed more engagement with technology than older adults with MCI (44.8/60 and 35.5/60, respectively).

The CT serious game program called Kitchen and Cooking (Manera et al., 2015), was used with older people with MCI and Alzheimer’s disease obtaining positive usability results, since 70% of the tasks were successfully completed. UX results were also positive, because the participants were generally interested, did not get very tired, played freely for an average of 3 h 48 min and performed 85% of the scenarios at home. In addition, they were motivated (intrinsic motivation being higher than extrinsic), felt more positive emotions than negative ones and were highly satisfied (people with Alzheimer disease showed more satisfaction than those with MCI).

The eWALL platform (Kyriazakos et al., 2017) provides CT in video game format and also includes home assisted environment functions. Only UX was measured, the results showing that MCI participants preferred cognitive exercise, activity and sleep apps. Finally, in another study (Garcia-Sanjuan et al., 2017), older adults with MCI, severe cognitive impairment and HE received CT in game format through the Tangibot robot. Usability measures showed that subjects with severe cognitive impairment had more difficulties, completed fewer tasks, made more mistakes and needed more help from the moderator. However, the time they spent on completing the tasks was similar to that spent by people with MCI, and 57.5% of the participants needed help. On the other hand, HE performed fewer unnecessary actions, took less time to complete the tasks, and completed more tasks than older adults with MCI. Regarding the latter, the difference was not significant, and neither was the fact that they made more mistakes than individuals with MCI. Overall UX results were positive: 62.5% expressed enjoyment and 80% liked it, although HE showed it more than individuals with cognitive impairment.

## Discussion

This systematic review presents current measures and characteristics of usability and UX in the field of CS, CT and CR technologies for older adults with MCI or dementia. This is relevant because of the importance of developing cognitive intervention systems in a digital age where technology is required to cater for the needs and particularities of this population group. A total of 10 studies were selected: five aimed at CS and five at CT. Most of them used a serious game format (*n* = 6), while the others consisted of cognitive exercises. The prevalence of the serious game format is consistent with other studies that support it as an increasingly popular alternative for the treatment of cognitive impairment because of its contribution to user motivation (Johnson et al., 2016; Manera et al., 2017). In addition, most were fundamentally computer-based programs (*n* = 6), which is also consistent with other studies where this hardware is found to facilitate older adults’ interaction with technology (Góngora Alonso et al., 2019).

Given the ongoing debate on the relationship between usability and UX as broad terms that can overlap, this review took the position of considering UX (which focuses on the subjective area) as an extension of usability (which also evaluates objective items) (Tractinsky, 2018; Sauer et al., 2020). This allowed a consistent categorization in the different studies found where each had its own position. Both usability and UX were measured in 6 of the studies, while the other four focused on UX.

Effectiveness and efficiency appeared in the studies among the measurement of objective aspects of usability. Effectiveness consisted of counting the number of tasks that were completed by the participants (*n* = 4) and the number of mistakes (*n* = 2). According to Georgsson and Staggers (2016), these are the most commonly used effectiveness measures to provide information on how easy or difficult it was for subjects to solve them and of the obstacles that hindered their progress. However, apart from knowing whether the individual managed to complete the task, the resources he/she used are also important (International Organization for Standardization [ISO], 2019b), which is where efficiency comes into play. In the studies found, efficiency involved the time spent by participants on completing the tasks (*n* = 4), the number of unnecessary actions (*n* = 1) and moderator assistance (*n* = 3). These measures are consistent with other investigations (Landman et al., 2014; Bevan and Carter, 2016) where time spent on task completion is commonly used (Georgsson and Staggers, 2016).

Regarding UX, which allows the measurement of subjective aspects of usability, almost all the studies found used questionnaires and scales (*n* = 8). According to Albert and Tullis (2013), these self-reporting data collection tools provide the most relevant information about users’ perceptions: if they report positive feelings or reactions about a technology, then they are likely to use it or reuse it. Some of the scales and questionnaires in the review were already standardized (e.g., TAM, PANAS, UEQ). Carvajal et al. (2011) emphasizes the use of standardized tools as a means of ensuring measuring quality, since they are valid, reliable, precise and feasible. The standardized and non-standardized questionnaires/scales found were not only about satisfaction, but encompassed other dimensions such as acceptability, which according to Borsci et al. (2020) is related, as well as other elements that are considered to be part of UX such as motivation, affectivity, perceptions and sustainability (Kramer, 2012; Lallemand et al., 2015).

The other tools found to measure UX consisted of observing participants’ behavior while they interacted with the cognitive intervention technologies and conducting interviews. According to Hartson (2019), both are necessary, because they are a way of gathering information about what subjects express in their verbal and non-verbal behavior, as well as what they report. In addition, other measures of UX found were time spent and number of tasks that participants performed freely at home as often as they wanted, alongside attendance rates when tasks were performed at a center. These indicators of how often a program is used are linked to UX and are indicative of whether or not the technology system will be successfully implemented (Hong et al., 2014; Partala and Saari, 2015).

On the other hand, the study by Haesner et al. (2015) was different from the others in this review, because these authors only presented evidence of already existing technology and CT program exercises to gather information and develop their own software in the future. Although it could be argued that preferences and attitudes not linked to a current experience are not considered UX (Albert and Tullis, 2013), such criticism does not apply in this case, since UX can involve indirect interaction, which can trigger a certain behavior, and because of the effect of observing and thinking about the system, product or service (Albert and Tullis, 2013; Hartson, 2019).

Furthermore, because effectiveness and efficiency focus on objective aspects of usability while UX focuses on subjective aspects such as satisfaction, it is important to be aware of the differences and similarities in the studies’ measurement results. According to Pluye et al. (2009), although quantitative and qualitative data may seem divergent, they actually have great potential to improve assessment and understanding of the topic in question.

In the studies of CS and CT technologies found (González-Palau et al., 2013; Ben-Sadoun et al., 2016; Garcia-Sanjuan et al., 2017; Tziraki et al., 2017), usability measures were consistent in that the performance of older adults with dementia or MCI was poorer (they completed fewer tasks, took longer to complete them or needed more help) as compared to that of healthy elderly people, which was also observed in other studies on their general functioning (de Frias et al., 2009). If both population groups are compared, usability results may appear negative for individuals with cognitive impairment; however, if attention is paid to the latter’s performance, it can be observed that, despite their difficulties, they were able to complete a considerable number of tasks, reduce their errors and time spent on task completion, and also lengthen their period of interaction with the program. These results can also be seen in the studies found that only included individuals with cognitive impairment in their measurements (Boulay et al., 2011; Manera et al., 2015), and are in line with Holthe et al. (2018), who stress that technology programs with adequate usability allow people with lower cognitive capacity to achieve goals because they are user-friendly.

It is also essential to know how older adults with MCI or dementia regard their experience with the relevant technological intervention, given that it will help to improve its usability and success (Foster, 2014; Holthe et al., 2018). Regarding the measurements of UX reported in the review, in some studies people with cognitive impairment perceived difficulties in using the technological tool without help or in using certain icons and devices (González-Abraldes et al., 2010; González-Palau et al., 2013; Ben-Sadoun et al., 2016). These subjective data results are related to the objective performance data described above. However, these difficulties did not prevent them from having a positive experience, and, according to most of the studies they reported feelings of satisfaction, fun, engagement, interest, motivation, acceptability, a desire to continue using it as often as they wished, and even an increase over time in their feeling of self-efficacy (González-Abraldes et al., 2010; Boulay et al., 2011; González-Palau et al., 2013; Haesner et al., 2015; Manera et al., 2015; Ben-Sadoun et al., 2016; Djabelkhir et al., 2017; Garcia-Sanjuan et al., 2017; Kyriazakos et al., 2017; Tziraki et al., 2017).

These positive results are further supported by the low number of dropouts reported in the studies, most of which were due to medical problems associated with old age and aspects that were beyond the subjects’ control. Torous et al. (2020) argue that UX and dropouts are strongly related, and that the degree of dropouts depends on how the technology was introduced. In this regard, most of the studies in the review included a period for older adults to become familiar with the cognitive intervention tool. On the other hand, half of the technological tools contained other functions besides CT, which could have influenced the results of the usability and UX measurements obtained. In fact, Contreras-Somoza et al. (2020) indicate that complementing cognitive intervention systems with social or emotional functions could improve adherence.

Finally, it is important to consider this review’s limitations. First, most of the studies were conducted in Europe and most of the participants were women. As is known, sex and social background influence a person’s characteristics (McCarrey et al., 2016). Second, no studies were found on usability or UX of CR technology, perhaps because CR focuses on improving functionality in activities of daily living, i.e., it is not restricted to cognitive tasks (Oltra-Cucarella et al., 2018). Third, some studies had few participants with dementia or MCI, although they equally provide relevant data in a field where few investigations were found. However, studies on participatory design or user-centered design could provide more data, even if they do not focus specifically on usability and UX evaluation. Computer sciences and engineering publications could also provide data in this area, although these sciences do not usually approach it from a clinical perspective. Fourth, not all studies provided detailed information about their software and hardware, as did other studies (Irazoki et al., 2020); nevertheless, for the purposes of this review they gave an overview of the technology used. Fifth, another limitation is not having assessed the risk of bias and the quality of the studies; however, only articles published in scientific journals were used. Finally, comparing instruments that measure usability and UX was challenging, because there is no consensus on these broad terms (Sauer et al., 2020) and there are also few studies involving older people with dementia or MCI, which may be due to a certain skepticism about the level of feedback they can provide and doubts about the appropriateness of testing prototypes on them, since possible mistakes can make them feel confused and disappointed with new technologies (Boman et al., 2014; Holthe et al., 2018).

## Conclusion

This systematic review identified 10 studies that measured usability and UX in cognitive intervention technologies for older people with dementia or MCI. The studies showed lack of scientific consensus on the relationship between usability and UX, most of them using measures indiscriminately. This review’s approach to UX (which focuses on subjective data) as an extension of usability (which also evaluates objective data) made it possible to consistently categorize the tools used to measure these parameters.

The objective measurement of usability, efficiency and effectiveness data led to the conclusion that older adults with cognitive impairment can complete a considerable number of tasks, even though they require more time and help in technological cognitive interventions. Likewise, questionnaires and scales were the most widely used tools to measure the subjective data of satisfaction and its related dimensions, the results showing that they regarded it as a positive experience, despite certain difficulties involving elements of the interface or the devices.

Measuring usability and UX in cognitive intervention technologies for older adults with MCI or dementia provides an integrated view that can contribute toward their proper development, since it is not only important to know if the technology is easy to use to achieve the therapeutic goals, but also whether the user perceives it as pleasant. To take these measurements it is essential to involve the target population: older people with cognitive impairment, who can give valuable feedback, despite their difficulties.

For future work, more research is needed to include this population group in usability and UX studies, as well as standardized tools and consensus on the relationship of these terms, which are crucial to guarantee the future effectiveness and success of technological interventions in the field of CS, CT, and CR. In this sense, it would also be interesting to compare the usability and UX results with effectiveness results. Finally, it is also necessary that studies give more information about the software and hardware features in order to have a more enriching view of usability and UX measures.

## Data Availability Statement

The raw data supporting the conclusions of this article will be made available by the authors, without undue reservation.

## Author Contributions

L-CS and MF-M conceived the presented systematic review. LC-S, EI, and AD-B performed the data collection and were supervised by JT-G. LC-S drafted the manuscript with feedback of JT-G and IT-D. MP-B and EP-V provided the further critical feedback. MF-M, JT-G, and LC-S revised the last version of the manuscript. All the authors contributed to the manuscript and approved the submitted version.

## Conflict of Interest

The authors declare that the research was conducted in the absence of any commercial or financial relationships that could be construed as a potential conflict of interest. The handling editor declared a shared affiliation with several of the authors EI, AD-B, MP-B at time of review.

## Footnotes

1 https://www.crd.york.ac.uk/prospero/display_record.php?ID=CRD42020158147
